# The involvement of cyclotides in mutual interactions of violets and the two-spotted spider mite

**DOI:** 10.1038/s41598-022-05461-y

**Published:** 2022-02-03

**Authors:** Blazej Slazak, Aleksandra Jędrzejska, Bogna Badyra, Anna Sybilska, Mariusz Lewandowski, Marcin Kozak, Małgorzata Kapusta, Reza Shariatgorji, Anna Nilsson, Per E. Andrén, Ulf Göransson, Małgorzata Kiełkiewicz

**Affiliations:** 1 W. Szafer Institute of Botany of the Polish Academy of Sciences, 46 Lubicz, 31-512 Cracow, Poland; 2 Pharmacognosy, Department of Pharmaceutical Biosciences, Uppsala University, Box 574, 751 23 Uppsala, Sweden; 3 Laboratory of Neurobiology, Nencki-EMBL Center of Excellence for Neural Plasticity and Brain Disorders: BRAINCITY, Nencki Institute of Experimental Biology of the Polish Academy of Sciences, 02-093 Warsaw, Poland; 4 Institute of Horticulture Sciences, Department of Plant Protection, Section of Applied Entomology, Warsaw, University of Life Sciences-SGGW, 159 Nowoursynowska, 02-776 Warsaw, Poland; 5 University of Information Technology and Management in Rzeszów, 2 Sucharskiego, 35-225 Rzeszow, Poland; 6 Department of Plant Cytology and Embryology, Faculty of Biology, University of Gdańsk, 59 Wita Stwosza, 80-308 Gdańsk, Poland; 7 Spatial Mass Spectrometry, Science for Life Laboratory, Dept. of Pharmaceutical Biosciences, Uppsala University, Box 591, 751 24 Uppsala, Sweden

**Keywords:** Plant ecology, Plant stress responses, Chemical biology, Chemical ecology, Peptides

## Abstract

Plants employ different chemicals to protect themselves from herbivory. These defenses may be constitutive or triggered by stress. The chemicals can be toxic, act as repellents, phagosuppressants and/or phago-deterrents. The two-spotted spider mite (*Tetranychus urticae*) is a generalist arthropod herbivorous pest and its feeding causes extensive damage both to crops and wild plants. Cyclotides are cyclic peptides involved in host-plant defenses. A single *Viola* sp*.* can produce more than a hundred cyclotides with different biological activities and roles. The organ and tissue specific cyclotide patterns change over the seasons and/or with environment, but the role of biotic/abiotic stress in shaping them remains unclear. Here, we demonstrate the involvement of cyclotides in mutual interactions between violets and mites. We used immunohistochemistry and mass spectrometry imaging to show the ingested cyclotides in *T. urticae* and assess the *Viola odorata* response to mite feeding. Moreover, to assess how mites are affected by feeding on violets, acceptance and reproductive performance was compared between *Viola uliginosa*, *V. odorata* and *Phaseolus vulgaris.* We demonstrate that cyclotides had been taken in by mites feeding on the violets. The ingested peptides were found in contact with epithelial cells of the mite digestive system, in the fecal matter, feces, ovary and eggs. Mites preferred common bean plants (*P. vulgaris*) to any of the violet species; the latter affected their reproductive performance. The production of particular cyclotides in *V. odorata* (denoted by molecular weights: 2979, 3001, 3017, 3068, 3084, 3123) was activated by mite feeding and their levels were significantly elevated compared to the control after 5 and 21 days of infestation. Specific cyclotides may affect mites by being indigestible or through direct interaction with cells in the mite digestive tract and reproductive organs. A group of particular peptides in *V. odorata* appears to be involved in defense response against herbivores.

## Introduction

Plants in general utilize various phytochemical strategies to withstand or defend themselves from attacks of phytophagous arthropods. These strategies may affect the behavior (antixenosis) and performance (antibiosis) of herbivores, as well as various compensatory/recovery mechanisms (tolerance)^1–4^. A variety of bioactive chemical compounds (allelochemicals) may be constantly produced to provide non-specific protection, or their synthesis might be induced in direct response to herbivory^5–7^. Those, repellent, phagosuppressant, phagodeterrent or toxic chemicals that can effectively lower herbivore fitness and performance are important factors in a plant’s defensive armory^6,8^.

The two-spotted spider mite *Tetranychus urticae* Koch (Acariformes: Trombidiformes: Tetranychidae) is a cell-sucking arthropod pest with a worldwide distribution and as a generalist able to feed on a wide range of host-plants^9^. However, host-plant nutritional quality and different allelochemicals can strongly affect mite developmental and reproductive potential^10–14^. *T. urticae* is able to evade many chemical defenses of its host plants by utilizing various detoxifying enzymes such as P450 monooxygenases, carboxyl/cholinesterases and glutathione-*S*-transferases^15–17^. *T. urticae* can also avoid feeding on plant tissues that produce defensive compounds or it can sequester them^14,16^. Other mechanisms of mite resistance are based on the expression of ATP-binding cassette transporters or the development of target site mutations^16–18^. Therefore, irrespective of which plant species/cultivars *T. urticae* colonizes—whether wild plants, economically important crop plants—in most cases its feeding causes extensive damage and loss of yield^10,19^.

Plant species belonging to the genus *Viola* (Violaceae) produce cyclotides—head-to-tail cyclic peptides, composed of about 30 amino acids^20^. The peptides include cysteines in conserved positions, which form three disulphide bonds in a knotted conformation^21^. This structure—the cyclic cystine knot—gives the molecule some unique physicochemical properties. Cyclotides are exceptionally resistant to enzymatic (e.g. proteolytic), chemical and thermal degradation^40^. Besides the Violaceous family, in which the peptides seem to be produced by most species, cyclotides have been found in members of the Rubiaceae, Cucurbitaceae, Fabaceae, Solanaceae and Poaceae families^22–28^.

Plants from the above mentioned botanical families produce mixtures of dozens, or in some cases, more than a hundred different cyclotides with different biological activities and roles^25,29^, many of which are considered to be host-plant defense molecules^30^. These peptides have been reported as possessing antimicrobial properties and having detrimental effects on some chewing insect larvae^30–32^. The distribution of particular peptides among organs and tissues is seemingly linked to their targets and biology. For example, peptides active against nematodes tend to be produced in the roots, whereas the antifungal peptides are found in the leaf epidermis^32–34^. The cyclotide pattern in the plant changes over the seasons and varies in different environments but the factors influencing it are largely unknown^35,36^.

Recently, we have shown that some cyclotides present in violets, namely cycloviolacins, are active against a phloem-sucking insect pest, the green peach aphid (*Myzus persicae*, Sulzer)^37^. Whether consuming the violetes might act against cell-feeding mite herbivores and whether violets exposed to mite attack can induce responses involving cyclotides, remains unknown. The present study was therefore undertaken to investigate different aspects of the *Viola*–*T. urticae* interactions. Specifically, we examined whether cyclotides are ingested when mites feed on violets and how consuming the diet composed of violet’s cell content may affect the mite digestive system and reproductive potential. Finally, we assess whether the violets exposed to mites respond in terms of cyclotide production and composition.

## Results

### Cyclotides in mite-damaged leaf tissues, mite body, eggs and feces

**Figure 1:**
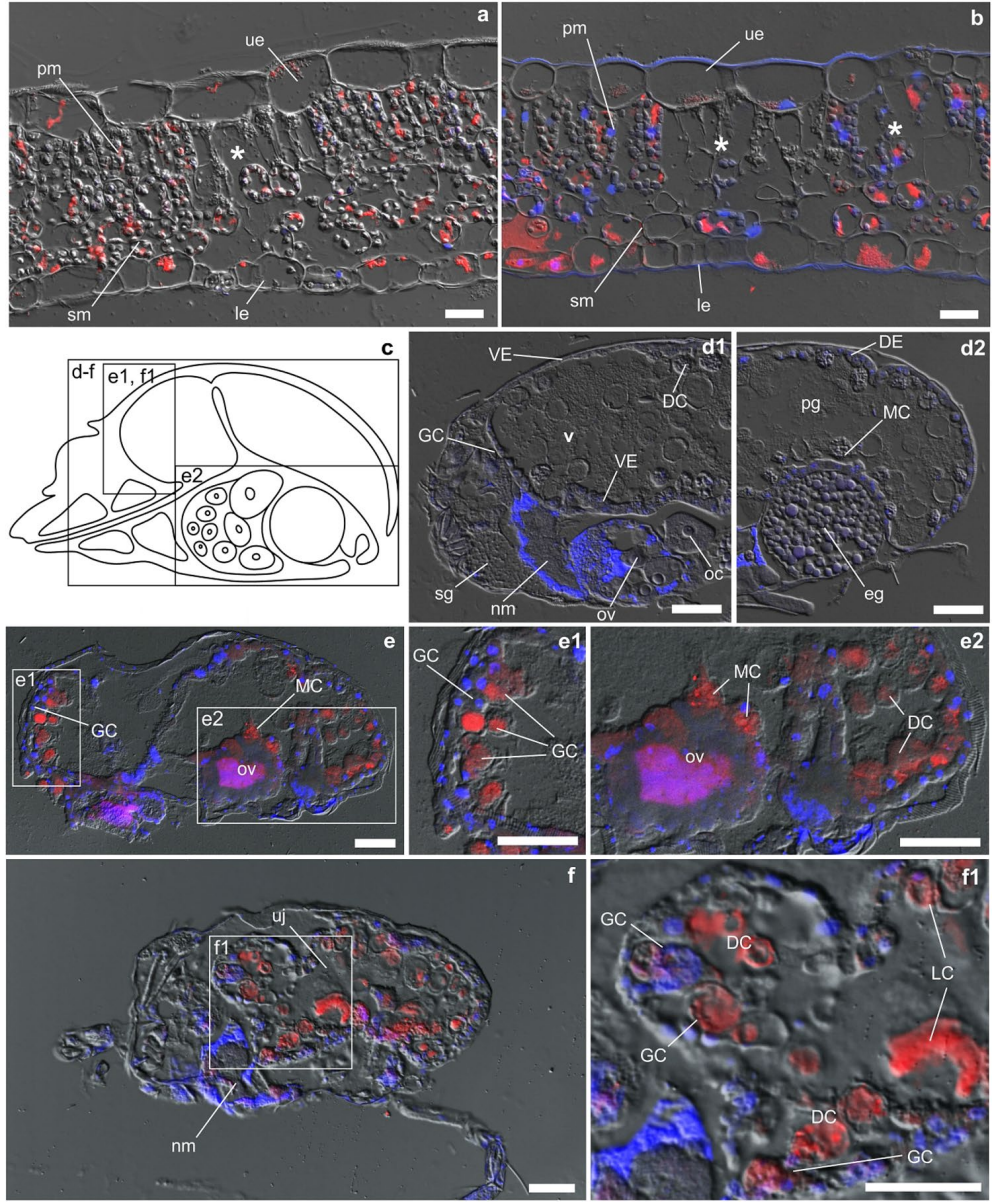
Immunolocalization of cyclotides in mite-damaged leaf tissues and in mites after ingestion. (**a**) Transverse sections of mite-infested leaves of *V. uliginosa*, and (**b**) *V. odorata* stained with immunohistochemistry. Cyclotides, found in the lower (le) and upper (ue) epidermis, palisade (pm) and spongy mesophyll (sm), are indicated in red; nuclei and chloroplasts are in blue. In the case of both *Viola* species, leaf injuries caused by mite feeding were localized in the palisade and spongy mesophyll (indicated with asterisks). (**c**) Simplified scheme of TSSM female anatomy. (**d**,**e**,**f**), Transections of different female mites fed on bean (control) (**d1** and **d2**); and cyclotide-challenged females fed on *V. odorata* (**e**,**e1**,**e2**,**f1**,**f2**). Different tissues and structures and tissues can be distinguished: salivary gland (sg); nervous tissues or central nervous mass (nm); ovary (ov) with oocytes (OOCs) and egg (eg) adjacent to the ventriculus (v) and posterior midgut (pm); lumen of digestive tract filled with digestive cells (DCs) or floating cells originating mainly from the midgut epithelium and comprising phagocytes containing a bolus of food; generative cell (GCs) and lateral cell (LCs) cells located within caeca as well as from microvilli epithelial cells (MCs) located within the posterior midgut. DCs with undigested plant material containing cyclotides, to be finally extracted as fecal pellets are located in the rectum (r). Ventricular epithelium (VE) is located within the dorsal and ventral parts of the ventriculus, whereas the dorsal midgut is lined with dorsal epithelium (DE). Cyclotides, indicated with red fluorescence, are in close proximity to the epithelial cells (GCs, LCs, MCs), digestive cells (DCs) and ovary (ov). Abbreviations of mite anatomical structures adapted from Bensoussan et al.^60^. Scale bars: a, b = 25 µm; d-f = 50 µm.

Transverse sections of the non-infested *V. uliginosa* and *V. odorata* control leaves revealed an internal structure of mesophyll tissues typical of dicots. In *T. urticae* infested plants, cyclotides were detected using immunohistochemistry within the central vacuoles of palisade and spongy mesophyll cells, as well as within the cells of the lower and upper epidermis (Fig. 1a,b). Mites were found to have fed on the cyclotide-containing cells of the leaf mesophyll. Empty palisade and spongy mesophyll cells with collapsed and/or misshapen walls and enlarged intercellular spaces were clearly visible as evidence of mite feeding activity in mite-infested leaves of both *V. odorata* and *V. uliginosa* (Fig. 1a,b).

**Figure 2:**
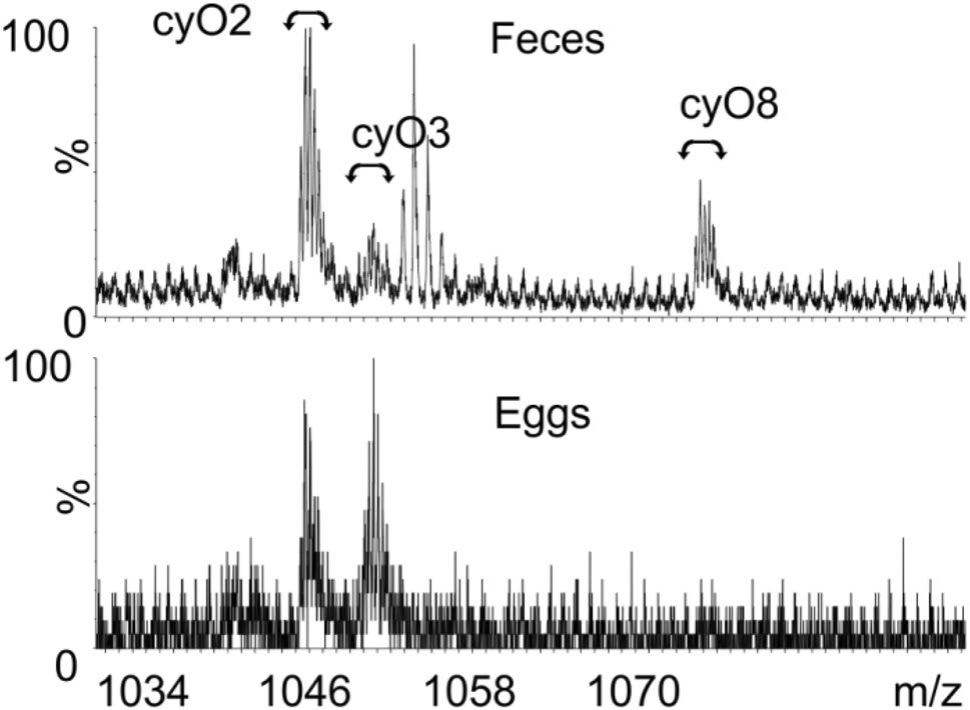
LC–MS analysis of extracts prepared from mite feces and eggs collected after 2 weeks of TSSM female feeding on *V. odorata*. The 3 + peaks corresponding to three (cyO2, cyO3 and cyO8) and two (cyO2, cyO3) different cyclotides were found in the mass spectra of the feces and egg extracts, respectively.

Immunohistochemistry analysis allowed to track cyclotides in the mite body (schematic representation in Fig. 1c). No fluorescent immunohistochemical signal was detected in sections of mites that had fed on bean plants, thus proving the specificity of anti-cycloviolacin antibodies (Fig. 1d). Immunohistochemical analysis of mites that had fed upon *V. odorata* revealed that, following ingestion, the plant material containing cyclotides moved to the midgut (Fig. 1e,e1,f,f1). Cyclotides were detected in the different types of epithelial cells of the ventriculus, midgut and posterior midgut, as well as in the large vesicles of free-floating phagocytes within the lumen of lateral caeca, and within fecal pellets ready to be excreted from the rectum (Fig. 1e,e2). Cyclotides were also detected within the ovary (Fig. 1e,e2). LC–MS analysis of extracts from the eggs of mites that had fed upon *V. odorata* showed the presence of only cyO2 and cyO3, whereas cyO2, cyO3 and cyO8 cyclotides were detected in the feces (Fig. 2).

### Plant response to mite feeding in terms of cyclotide production

**Figure 3:**
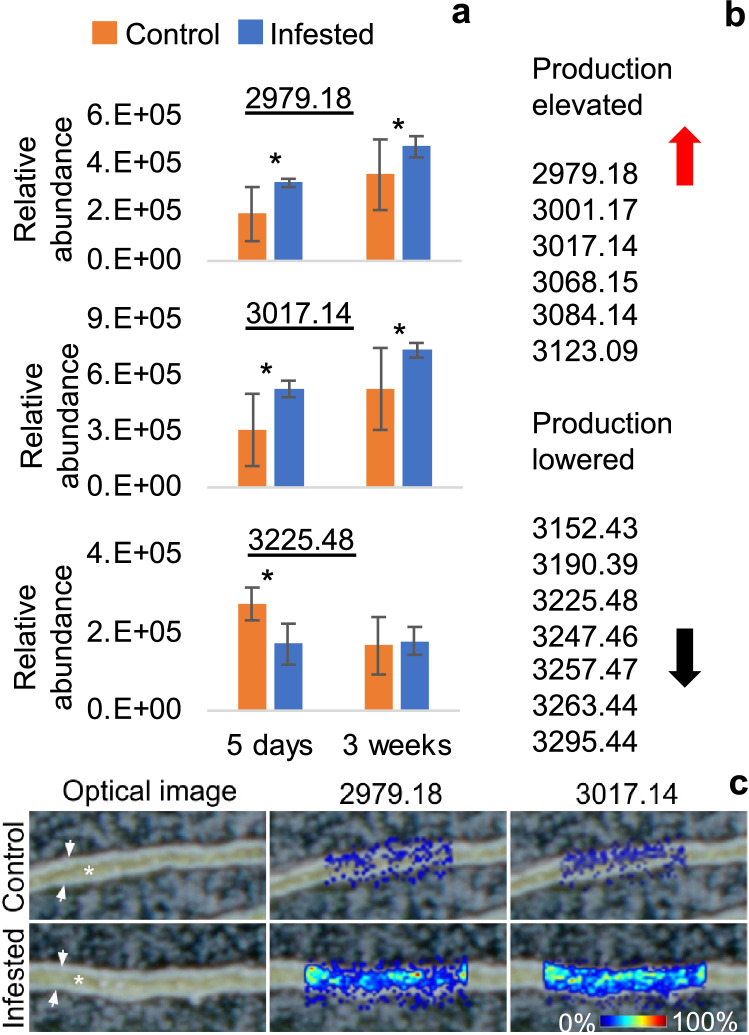
*V. odorata* response to mite feeding in terms of cyclotide production: (**a**) Comparison of mean relative quantities of example peptides denoted with their monoisotopic molecular masses, between infested and control plants, 5 days and 3 weeks after infestation. Asterisks indicate statistical significance at p < 0.05 by ANOVA and t-test. (**b**) The list of significantly (p < 0.05, ANOVA, t-test) more abundant (higher) or less abundant (lower) peptides in infested and control plants. (**c**) MALDI-MSI image showing particular peptides appearing in the leaf mesophyll in response to mite infestation—heat scale, the warmer color indicates higher abundance. Cyclotides are named according to their monoisotopic molecular masses.

Analysis of the relative abundance of cyclotides in *V. odorata* plants in response to mite feeding showed that the production of a whole group of specific peptides (denoted by their monoisotopic molecular masses: 2979, 3001, 3017, 3068, 3084, 3123) was increased in mite-infested plants compared with the control (Fig. 3a,b). The production levels of these peptides were higher in plants after 5 days and 3 weeks following infestation. The levels of other cyclotides appeared to be lower in the mite-infested plants (3152, 3190, 3225, 3247, 3257, 3263, 3295) compared to the control. However, these effects were apparent only after 5 days of mite infestation and there were no differences in the abundances of these peptides in mite-infested and control plants after 3 weeks (Fig. 3a,b). Matrix-assisted laser desorption/ionization mass spectrometry imaging (MALDI-MSI) analysis confirmed that the particular cyclotides that were produced at higher levels are accumulated in the *Viola* leaf mesophyll cells in response to mite infestation (Fig. 3c). The detailed quantitative comparisons of cyclotide content in mite-infested plants and controls with mean values and calculated levels of statistical relevance are given in Supplement 1.

### Greenhouse *T. urticae* choice assay between *V. odorata* and *V. uliginosa*

**Figure 4:**
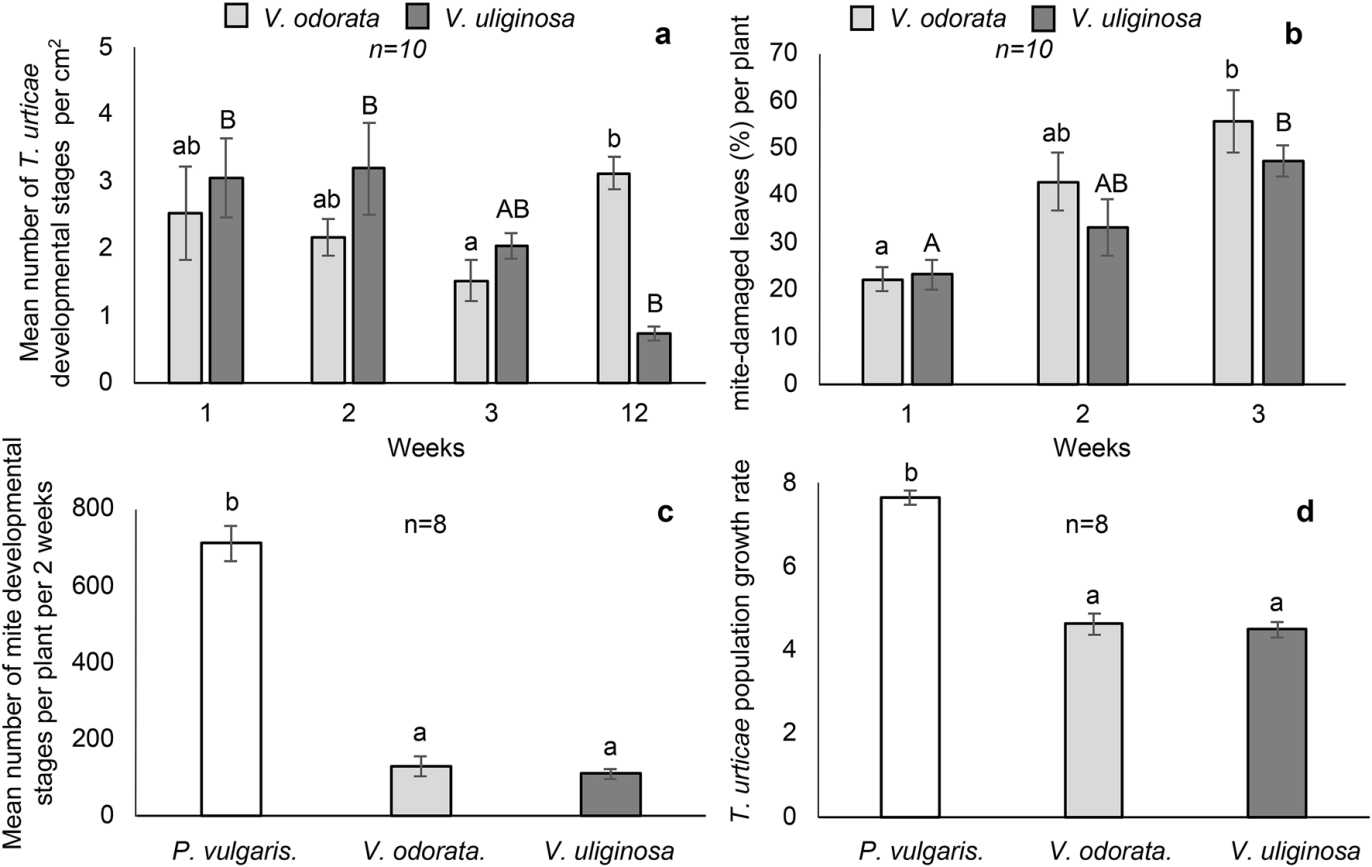
Effect of host plant (*V. odorata*, *V. uliginosa*, *P. vulgaris*) on *T. urticae* mite reproductive potential and feeding (green house experiments): (**a**) mean number of all *T. urticae* developmental stages per cm^2^ per leaf 12 weeks after infestation. (**b**) Percentage of *T. urticae*-injured leaves per plant 3 weeks after infestation. (**c**) Mean number of all *T. urticae* developmental stages per plant per 2 weeks (**d**) *T. urticae* population growth rate (PGR) 2 weeks after plant infestation. Mean values followed by different lowercase or uppercase letters are significantly (p < 0.05) different by ANOVA and Tukey’s honestly significant difference test.

Mites were able to colonize both *V. odorata* and *V. uliginosa* when given the choice under greenhouse conditions. Following the first 3 weeks of infestation, the population remained at the similar level on both *Viola* species (average 3.5 developmental stages per cm^2^ of leaf) (Fig. 4a). The percentage of mite-damaged leaves within each plant increased from an average of 22% to an average of 52% after the 1st and 3rd week of infestation, respectively (Fig. 4b). The extent of leaf damage did not vary significantly between the two *Viola* species. Nine weeks after the initial colonization, the number of mobile stages and eggs decreased drastically on leaves of *V. uliginosa* whereas it remained unchanged on *V. odorata* leaves (Fig. 4a). The number of mites in all developmental stages after 2 weeks for each of the two *Viola* plant species was almost one-seventh of that on common bean (Fig. 4c). Consequently, the population growth rate of mites on *Viola* plants was almost half of what was seen for common bean (Fig. 4d).

### Greenhouse assay on effect of host plant (V. odorata, V. uliginosa, P. vulgaris) on T. urticae mite reproductive potential

The developmental time of mite juvenile stages (egg-to-adult) was significantly longer for specimens feeding on *V. odorata* than on either *V. uliginosa* or bean plants, except the duration of the egg stage (Table 1). However, the survival rate of immatures did not differ between the three host plants. Female longevity differed significantly depending on the host-plant they fed upon (Table 1). Females that fed on the *V. uliginosa* lived the longest, while the lifetime of the females that fed on bean was the shortest. The total mite fecundity, similarly to daily fecundity, differed significantly among all studied host-plants. On beans, it was actually a few times higher than it was for females that fed on either of the *Viola* species (Table 1).

**Table 1:** The effect of the host-plant on the mean duration of the *T. urticae* developmental stages and different reproductive parameters.

	*P. vulgaris*	n	*V. odorata*	n	*V. uliginosa*	n	p-value
**Immature development times and survival rates**							
Egg (days)	4.2 a	49	4.4 a	52	4.2 a	49	0.256
Egg to adult (days)	9.3 a	43	10.2 b	46	9.3 a	43	0.001
Survival rate of immatures	0.86 a	50	0.85 a	54	0.81 a	54	0.797
**Reproductive parameters of females**							
Female longevity (days)	9.4 a	30	10.7 b	27	11.8 c	36	< 0.001
Total fecundity (eggs per female)	113.9 a		32.6 b		10.8 c		< 0.001
Daily fecundity (eggs per female per day)	9.1 a		2.3 b		0.8 c		< 0.001

Different lowercase letters within rows indicate the means to be significantly different (p < 0.05); n = number of replicates.

Life-history data obtained from daily observations were used to construct life tables (Table 2). All calculated parameters were highest for the population that was reared on bean leaves. The *Viola* plants apparently provided a deficient diet—the intrinsic rate of population increase (*r*_*m*_) of mites was more than twice as high on bean plants. Net reproductive rate (*R*_*0*_), intrinsic rate of population increase (*r*_*m*_) and the finite rate of population increase (*λ*) did not differ significantly between populations fed on either species of *Viola*. Such difference was noted only for mean generation time (*T*), which was shortest for the population reared on *V. uliginosa* (Table 2).

**Table 2:** The comparison of *T. urticae* population parameters on different host-plants.

Population parameter (SE)	*P. vulgaris*	*V. odorata*	*V. uliginosa*
Net reproductive rate (*R*_0_)	51.8 (4.18) a	6.9 (0.98) b	4.9 (0.86) b
Mean generation time (*T*)	14.4 (0.35) a	16.6 (0.42) b	15.5 (0.32) c
Intrinsic rate of population increase (*r*_*m*_)	0.27 (0.0005) a	0.12 (0.0008) b	0.10 (0.0120) b
Finite rate of population increase (λ)	1.30 (0.0069) a	1.12 (0.0088) b	1.11 (0.0133) b

Standard errors are given in parentheses. Different lowercase letters within rows indicate significant differences between parameter estimates (p < 0.05).

## Discussion

The current study clearly showed that cyclotides are ingested by mites feeding on violets. The localization of cyclotides in the leaf tissues of both violet species assessed in the present work was similar to that described previously^32,38^. The peptides were present in the cells of leaf epidermis, palisade and spongy parenchyma, and vascular tissues. That mites do ingest *V. odorata* cyclotides was evidenced by feeding damage being found in leaf mesophyll tissues that contained the peptides, as well as by the immunohistochemical analysis providing direct evidence of the peptides within their digestive system (lumen of ventriculus, epithelial cells of the midgut, fecal pellets), ovary and eggs. Cyclotides in the digestive tract may affect nutrient digestion by impairing ventricular function. This in turn could decrease mites’ fitness and developmental potential, as observed on *V. odorata*. Indeed, it has previously been found that the insecticidal activity of kalata B1 and B2 to *Helicoverpa punctigera* (Insecta: Lepidoptera) caterpillars results from the midgut epithelium cell membranes being disrupted^30,39^. It is reasonable to assume that the effects of cyclotides on mites may be caused by similar mechanisms. On the other hand, cyclotides detected in the feces of the mites fed on *V. odorata* indicate that the peptides are not fully digestible. It is well documented, that cyclotides, with their cyclic cysteine knot molecular structure, are very stable and resistant to proteolysis and degradation^40^. Thus, it seems that quantities of certain nutrients encased in cyclotides may be unavailable to the mite. This is important because certain amino acids essential to mites, such as: arginine, histidine, isoleucine, leucine, lysine, phenylalanine, tyrosine, and valine, are present in *V. odorata* cyclotides^35,41^. Thus, even though mites may ingest the mesophyll cell content of violet plants, they may eventually become malnourished.

The detection of cyclotides in the ovary and eggs of mites fed on *V. odorata*, clearly indicates that these peptides are transported outside the midgut. It has been well documented that nutrients are supplied to the maturing eggs via a number of routes including the hemolymph, the midgut cells adjacent to the ovary, the nutritive or nurse cells of the ovary, pinocytotic activity of plasmalemma cells of the developmental egg stages (e.g. vitellogenic oocytes), as well as by microvilli transcellular transport^42–44^. It seems likely that cyclotides are transported to the ovary and maturing eggs in a similar way as other nutritional compounds. Such an hypothesis is supported by the results of a study by Mothes-Wagner^45^ which showed that the lateral midgut consists not only of the restorative epithelium but also the transporting epithelium, whereas the dorsal and posterior lateral epithelium contain cells promoting excretion transport. However, it would require further studies to determine how cyclotides move into the reproductive organs. Interestingly, the cyclotide composition of the mite egg extracts, indicated in the current LC–MS analysis, differed from the feces extract. The egg extract also appeared to contain only a few of the peptides present in *V. odorata*^46^. This may reflect varying physicochemical properties of cyclotides composed of different amino acid sequences and their consequent ability to permeate biological membranes^47^.

The results of the present study indicate that the cyclotide composition of *V. odorata* leaves can change in response to mite feeding. Some of the peptides were present at significantly higher levels in mite-infested *V. odorata*, compared to the non-infested control. This may reflect the plant’s induced cyclotide production in response to mite-feeding stress. It is still a matter of debate whether cyclotides belong to the constitutive/innate defense system or if their production is triggered as a mitigative response to stress. A previous study on the expression of several cyclotide genes in response to various kinds of stress in *Oldenlandia affinis* indicated that the peptides are part of an innate defense system^48^. However, contemporaneous studies on *O. affinis* cell suspensions demonstrated a significantly higher production of kB1 in cultures treated with the biological elicitor—chitosan, which would suggest an induced response^49^. It has also been shown by numerous authors that in various cyclotide producing plant species, the production of peptides can be seasonal, can be influenced in in vitro cultures by treatments with endogenous plant growth regulators, or can vary in plants of the same species from different habitats^29,35,36,50,51^. In recent work, we found only minor differences of cyclotide production patterns between different populations of *V. odorata* from the Canary Islands^52^. However, that study concentrated on the main cyclotide constituents in the plant. In the present study, the abundance of major cyclotides was not significantly different between the mite-infested *V. odorata* plants and the non-infested controls, suggesting that some cyclotides are produced constitutively, whereas others are activated and produced in response to specific mite-stress. Other cyclotides appeared to be down-regulated in mite-infested plants. This may reflect the loss of some pool of peptides from the tissues damaged by mite feeding. It may also indicate that upon certain type of mite stress, the plant allocates more resources into the production of specific peptides, whereas the accumulation of others, less critical, is halted. Recently, we have shown that cyclotides can be decomposed in the plant cell, but that different peptides are degraded at different rates^53^. A similar mechanism could be responsible for creating a certain cyclotide pattern in response to particular mite-stress.

In the current study, using the MALDI-MSI technique, we have shown that certain peptides are produced and accumulated in the *V. odorata* leaf mesophylls in response to mite feeding. In a previous study, we developed MALDI-MSI methods allowing us to visualize the distribution of different cyclotides in various plant organs and tissues. It was shown there, that peptides were produced and accumulated in specific tissues, in such a way that they were locked to their target microbial pathogens of insect herbivores^32^. Such a specific pattern of cyclotide production and accumulation was also observed in the present study, since those peptides that are produced in response to mite feeding do in fact appear in the leaf mesophyll on which the mite feeds.

The results of the present study show that *T. urticae* can colonize violets (*V. odorata* and *V. uliginosa*) naturally. However, over time the population density decreased on *V. uliginosa*, while it remained stable but at a low level on *V. odorata*. This effects may be attributed to the differences in chemical defenses (including cyclotides) deployed by *V. uliginosa* and *V. odorata*^46,50^. When comparing *T. urticae* performance, reproduction and life-history on violets and bean plants as hosts it was found that population growth rates and various performance and reproduction parameters were lower on *V. odorata* than on bean. From these results, we infer that the phytochemicals unique to a *Viola* diet^54^, affect mite performance and reproduction. One possible explanation could be that the defense molecules produced by violets have phagosuppressive effects. If that were so, mites feeding on violets would suffer from their nutritional requirements being limited by poor uptake from the plant or through an impaired digestive system. This hypothesis is supported by our observations that the developmental times of juvenile stages (except eggs) were significantly prolonged and female fecundity was lowered. The apparent starvation and impeded development occurred only in the active life stages i.e. after the resources available in the egg had been consumed and the mobile stages had started to feed on their own. Other studies have also reported the host-plant phytochemicals of various species/cultivars are among the most important plant factors affecting tetranychid mite performance and reproductive success^10,11,14^. Taking the results from the present study and earlier work into consideration, the observed effects of *Viola* diet may be partially attributed to cyclotides that violets produce. We currently showed that cyclotides are ingested from violets and may affect the mite digestive system and reproductive organs. Moreover, *V. odorata* and *V. uliginosa* are known to produce and accumulate diverse cyclotides in large quantities^46,50^. These peptides have been found to affect various herbivores in different ways. For example, Jennings et al.^30^ showed the detrimental effects of the kB1 cyclotide on the growth and development of *H. punctigera* larvae and suggested that cyclotides are host-plant defense peptides^30^. Similar activity was later shown for the kB2 cyclotide^55^. These cyclotides can be found in both *V. odorata* and *V. uliginosa*^46,50^. Other cyclotides from *V. odorata* are known to inhibit the growth and development of the golden apple snail (*Pomacea canaliculata* Lamarck (Gastropoda: Ampullariidae) with effectiveness comparable with the synthetic molluscicide metaldehyde^56^. Recently, we demonstrated the very strong phagosuppressive effects of cycloviolacin cyclotides (cyO2, cyO3, cyO19) from *V. odorata* on a phloem-feeding insect pest—the aphid *M. persicae*^37^. In most cases the detrimental effects of these peptides against different organisms was found to be mild^30,37,55,56^. We have shown in the present study that the effects of a diet containing cyclotides on mites are similar. This suggests that the host-plant defense chemistry of violets, including cyclotides, do not cause immediate toxicity or increased mortality, but rather that they influence the mites’ fitness and growth.

Given the results of the present study as well as previous work showing the activity of cyclotides against various herbivorous pests, it can be hypothesized that these peptides may be used in crop protection. However, using pure cyclotides directly, e.g. by spraying them over the fields, seems not feasible as obtaining them in large quantities through chemical synthesis is still too complicated and expensive^50^. On the other hand, cyclotide genes can be inserted to genetically modified crops, to obtain varieties more resistant against common pests. Moreover, instead of purified peptides, raw violet extracts containing cyclotides could be also used as biopesticides, in a similar fashion as proposed for products made of other cyclotide-producing plant—*Clitoria ternatea*^57^*.*

## Conclusions

The current study provides a new insight into *Viola*–*T. urticae* mutual interactions and broaden the knowledge on the potential and biological roles of specific cyclotides produced by violets. The study shows that cylotides are up-taken by mites that fed on the cyclotide-containing mesophyll cells of *V. odorata*. Cyclotides ingested from *V. odorata* were indicated along the mite digestive tract and in the fecal pellets which suggest that the peptides may impair the nutrient uptake by affecting epithelial cells in the digestive tract and/or by being indigestible. Cyclotides may also have some effects on mite development and reproductive potential as they were found to be transported to the ovary and eggs. The diet composed of violets have negative effects on mite performance and reproductive potential. Prominently, the study indicates a group of cyclotides that may play a role in plant defense response against herbivores in *V. odorata*, production of which is activated in response to mite feeding.

## Methods

All methods comply with the relevant local and national guidelines, regulations and legislation.

### Plant material

#### *Viola* plants growing conditions

Plants of *Viola odorata* L. and *V. uliginosa* Besser were obtained from the collection of Professor Elżbieta Kuta (Cracow-Ugorek, Poland). They were propagated vegetatively and cultivated in the garden soil consisting of natural peats (WOKAS S.A., Łosice, Poland) in 12 cm diameter pots. Potted plants of both species were randomly placed on greenhouse tables in semi-shade, in two chambers of the greenhouse of the Warsaw University of Life Sciences—SGGW (WULS-SGGW), Warsaw, Poland (52° 09′ 35.7″ N 21° 02′ 36.2″ E). Plants were grown under 14–16 h of natural light (600–800 µmol photons/m^2^/s) during the growing season. Six to eight-week-old plants were used to assess (1) host-plant colonization by the mite, (2) mite population development and reproductive potential, and (3) the presence of cyclotides in mite digestive tracts, eggs and feces as well as in mite-infested *Viola* leaf tissues.

#### Common bean growing conditions

Common bean (*Phaseolus vulgaris* L. cv. Ferrari, PNOS, Ożarów Mazowiecki, Poland) seeds were sown weekly to ensure plant availability for all experimental procedures. Bean plants were grown in the garden soil (WOKAS S.A., Łosice, Poland) in plastic pots, 12 cm diameter, in an environmental growth chamber (Sanyo MLR-350) under light intensity of 150 µmol photons/m^2^/s, at 23 ± 2 °C temperature, 65 ± 10% RH and 16L/8D photoperiod.

### *Tetranychus urticae* culture and age-synchronized mite stock colonies

*T. urticae* was originally collected from *Sambucus nigra* and the mite culture was reared on common bean plants under controlled conditions for many generations (for details see Barczak-Brzyżek et al.^58^). To establish an age-synchronized mite stock colony^59^ on bean plants, pairs of a female deutonymph and a male were selected from the mite culture and transferred onto common bean leaves detached from 3-week-old plants. The leaves were placed upside down on wet cotton in petri dishes (10 cm diameter) and kept in SANYO Plant Growth Chambers (MLR-350H) under controlled conditions (150 µmol photons/m^2^/s, 23 ± 2 °C temp., 65 ± 10% RH, 16L/8D photoperiod). After about 2 days *T. urticae* females started to lay eggs. Individual females were transferred to the fresh bean leaves and after 24 h they were removed. 24-h-old eggs were left to develop into the age-synchronized offspring on common bean plants under controlled conditions as described above. Mites were reared for at least 3 generations before 5-day-old females were used to assess (1) the effect of the host-plant on the mite growth potential (the ‘no-choice’ greenhouse bioassays) and in (2) the lab assessments comparing the effect of host-plants on mite developmental and reproduction parameters.

The females originating from the age-synchronized colony reared on common bean were also transferred to some young *V. odorata* and *V. uliginosa* plants. The mite-infested potted Viola plants were housed in a metal frame (12 × 12 × 30 cm) covered with 100 μm nylon mesh and grown separately at 23 ± 1 °C, 60–70% RH, 16/8 h photoperiod in SANYO Plant Growth Chambers (MLR-350H). Three *Viola*-adapted mite generations were developed.

### Presence of cyclotides in mite-infested *Viola* leaf tissues, mite body, eggs and feces: immunohistochemistry and LC–MS

Twenty-five female mites that had fed on *V. odorata* or control bean plants for 2 weeks were collected to analyze for cyclotides in mite body. Ten leaf segments cut from 10 mite-infested plants of *V. odorata* and *V. uliginosa* were collected. Standard immunohistochemical protocols were used to image cyclotides in the plant material^38^ and mite body^37^. All samples were fixed in 4% paraformaldehyde and 0.25% glutaraldehyde in phosphate buffered saline (PBS), dehydrated in an ethanol series, embedded in Steedman’s wax, sectioned and immunostained by applying anti-cycloviolacin antibodies^37,38^. Morphological features of mites follow Bensoussan et al.^60^.

Feces and eggs of mites feeding on *V. odorata* or bean plants were collected under a stereomicroscope Olympus SZH10 (31.5×–63× magnification) and placed in separate Eppendorf tubes. The samples were extracted with 100 µl of 30% acetonitrile (ACN), and 0.05% trifluoroacetic acid (TFA). After centrifugation, 2 µl of the supernatant were extracted and analyzed using nanoAcquity UPLC-QTof Micro (Waters, Milford, MA) using the standard setup previously described^61^. Cyclotides were identified by matching their molecular masses calculated from *m/z* of the corresponding ions in MS, and their retention times with those of *V. odorata*, as described by Ireland et al.^46^.

### Relative quantitation of cyclotides in mite-infested plants

A subset of *Viola* plants was prepared for quantification of cyclotides content by MALDI-MSI. To minimize variation caused by external factors, we used *V. odorata* plants that were collected from the same garden collection but maintained for about 6 months in a culture room with an artificial cool white fluorescent light source (20 μmol/m^2^/s), 16 h/8 h (day/night) photoperiod and temperature of 20 °C. All plants were kept in the same conditions until the start of the experiment and then randomly assigned to control or the mite-infested groups.

For quantitative experiments, 8 V*. odorata* plants infested with 5-day-old female mites originating from the age-synchronized mite population, and 8 non-infested plants as a control group, were placed in a culture chamber with a 16 h/8 h day/night photoperiod at 20 °C in separate mini-glasshouses. Single plants were considered as biological replicates. Two–three fully developed leaves of similar size (4–5 cm in long) from each plant were collected from both mite-infested and control (non-infested) plants 5 and 21 days after infestation, and freeze-dried. These experiments were performed April–May 2019. TissueLyser (Qiagen, Germantown, MD) was then used to pulverize the material from each plant of freeze-dried material for 1 min. at 25 Hz. 2–4 mg of the samples were subsequently extracted for 2.5 min. at 25 Hz in 200 µl of 30% ACN, and 0.05% TFA per mg of sample. Preliminary experiments with serial dilutions of extracts were performed to assess the maximum detection point of the MALDI-MS method. Finally, all the samples were diluted fivefold prior to MALDI-MS semi-quantitative analysis in order to place the intensity values for selected cyclotide ions in a linear range. 0.5 µl aliquots of the diluted extracts were spotted on the metal target plates and analyzed using previously developed protocols^52^. The plates were sprayed using 6 passes of 2,5-dihydroxybenzoic acid (DHB, 35 mg/ml in 50% ACN and 0.2% TFA) with a solvent flow rate of 70 μl/min, a spray head velocity of 1100 mm/min, and track spacing of 2.0 mm. Nitrogen at 6 psi was used as the nebulizing gas. All the spots were analyzed (imaged) with 200 µm lateral resolution, which equates to about 100 pixels per spot. The average intensity of cyclotide [M + H] ^+^ ions per pixel per spot conveyed the relative quantity. The ions were selected for analysis when the monoisotopic peak was distinguishable in the average mass spectra and its intensity was within the linear range of the extract dilution used. The cyclotides were detected and identified in the mass range of 2.8 to 3.8 kDa.

MALDI-MSI analysis was performed as previously described by Slazak et al.^32^. Samples were collected at the same times as the quantitative analysis described above. Small rectangular pieces of approx. 0.25 cm^2^ were cut from leaves of mite-infested and control (non-infested) *V. odorata* plants, embedded in gelatin, snap-frozen in liquid nitrogen, sectioned, mounted on indium-tin-oxide-coated glass slides and further coated with DHB matrix solution using the conditions described above. The sections were imaged with 20 µm lateral resolution.

All measurements and imaging were performed using a MALDI Fourier-transform ion cyclotron resonance (FTICR) mass spectrometer (solariX 7T-2ω, Bruker Daltonics, Bremen, Germany) equipped with a Smartbeam II 2 kHz laser, and operated in positive-ion mode. All spectra were normalized against the root mean square (RMS). The relative quantitative analysis and images were prepared in msiQuant software^62^.

### Colonization of *Viola* spp. by mites and assessment of plant damage

#### Bioassay under ‘free choice’ greenhouse conditions

Randomly arranged *V. odorata* and *V. uliginosa* plants (n = 10 for each species) at the 5–8 leaves growth stage were naturally colonized (‘free choice’ conditions) by *T. urticae* in one of the greenhouse chambers and separated from the non-infested plants. The differences between the two *Viola* species in terms of the mite colonization rate and acceptance were determined. The numbers of *T. urticae* mobile stages and eggs on *Viola* plants were counted on both leaf surfaces [lower and upper], at 1, 2, 3 and 12 weeks following the initial infestation by using a stereomicroscope (Olympus SZH10, 31.5×–63× magnification). Since the leaf area of violet leaves varied within each tested plant, mite abundance was expressed as the number of developmental stages per cm^2^ of the leaf. Leaf area was measured without detaching the mite damaged leaves from the plant, through carefully outlining them on paper. The leaf outlines and 10 cm^2^ templates from the same paper were then cut out and individually weighed using an electronic balance. The leaf area was estimated based on the mass of the actual leaf area divided by the mass of the template (10 cm^2^) and multiplied by 10.

The severity of damage of mite-infested *V. odorata* and *V. uliginosa* plants was assessed at 1, 2 and 3 weeks following initial infestation. The percentage of mite-infested leaves was calculated based on the formula: the number of mite-injured leaves per plant in the experiment, divided by the total number of leaves and multiplied by 100.

### Greenhouse assay on effect of host plant (*V. odorata, V. uliginosa, P. vulgaris*) on *T. urticae* mite reproductive potential

To compare mite reproductive potential depending on the host-plant mite population growth rate (PGR) was assessed under ‘no choice’ greenhouse conditions. *V. odorata*, *V. uliginosa* and common bean were compared. Three-week-old bean plants were used for the age-synchronized mite stock colony rearing and ‘no choice’ bioassays. Specimens from the 3rd generation of *T. urticae* were used in bioassays on mite development and reproductive potential (under lab conditions). Common bean (*P. vulgaris*) is regarded as the mite’s most acceptable host-plant. All plants in the experiment (n = 8 for each species) were artificially colonized with 5-day-old females originating from an age-synchronized *T. urticae* population maintained on *P. vulgaris* at 24 °C, 60–70% relative humidity (RH) and long-day photoperiod (16 h/8 h). Females were transferred to one of the leaves of each 3-week-old-plant. After 14 days of mite development, all leaves were detached from the examined plants and the number of mobile stages and eggs was counted using a stereomicroscope (Olympus SZH10, 31.5×–63× magnification). The mite population growth rate (PGR) index was calculated according to the formula: r = log_2_ [(n/n_0_) + 1], where n = number of progeny, and n_0_ = number of females used for infestation^63^.

### Laboratory assay on the effect of host plant (*V. odorata, V. uliginosa, P. vulgaris*) on *T. urticae* developmental time, sex ration and survival

Mites from the age-synchronized stock colony adopted to appropriate plant species, were maintained on detached leaves of two *Viola* species and *P. vulgaris* plants in order to study development and reproduction in the laboratory setup^64^. The developmental time of eggs and juvenile stages, survival rate and sex ratio (proportion of females in the adult population) were assessed on detached leaves of 2-month-old *V. odorata* and *V. uliginosa* plants and 4-week-old bean plants. All leaves were placed lower side upwards on wet cotton wool in Petri dishes (5 cm diameter) according to the method described by Tomczyk and Kropczynska^10^. One female mite was placed on each leaf for 24 h to lay eggs. Females were then removed and only one egg was left on each leaf, which was then used as a rearing arena. The leaf surfaces were monitored every 24 h and the developmental stage reached was noted. The experiments were conducted in 50 replicates for each plant species.

To assess mite fecundity, female deutonymphs and males (originating from the age-synchronized stock colony adopted to appropriate *Viola* species or *P. vulgaris*) were paired up and transferred to 40 leaves in Petri dishes, one pair to a single leaf. The number of eggs laid by a single female was recorded and the eggs were removed daily until the death of the female. To keep leaves in good condition and protect females from escaping, the cotton wool on which leaves were kept was watered every day. All experiments were conducted in SANYO Plant Growth Chambers (MLR-350H) under conditions described previously.

Life tables were constructed from the observed age-specific survival rate (l_x_) and specific fecundity rate (m_x_) [net reproductive rate (R_0_), mean generation time (T), intrinsic rate of population increase (r_m_) and finite rate of population increase (λ)]^65,66^.

### Statistical analysis

The data on the mite development under ‘free choice’ and ‘no choice’ conditions were analyzed using one-way ANOVA and a post hoc Tukey’s honestly significant difference test. The data were considered significantly different if p < 0.05.

Since the immature development of mites on the 3 host-plants studied was represented by counts (i.e., the number of individuals), the data were analyzed using a general linear model with a Poisson distribution of residuals as a log function. Since the variability in the data when compared to the mean of the Poisson distribution was too small, quasi-generalized linear modelling was employed. Survival rate of immature stages (egg to adult) on the three host-plants was compared using a generalized linear model, with a binomial distribution of errors. Where we provide back-transformed parameter estimates we do not include standard errors since generalized linear models estimate SEs after transformation and they cannot be back-transformed. To compare the life-table parameters between the pairs of the three host-plants, pair-wise comparisons of these parameters were applied using the jackknife method^66^, without adjustment for multiple testing^67^.

The average relative quantities of particular cyclotides (average intensity per pixel in MALDI-MS) from the mite-infested and control plants were compared using one-way ANOVA with t-test and considered significantly different if p < 0.05.

## Supplementary Information

Supplementary Information.
